# Impact of substandard and falsified antimalarials in Zambia: application of the SAFARI model

**DOI:** 10.1186/s12889-020-08852-w

**Published:** 2020-07-09

**Authors:** Kathryn D. Jackson, Colleen R. Higgins, Sarah K. Laing, Chiluba Mwila, Tamaki Kobayashi, Matthew M. Ippolito, Sean Sylvia, Sachiko Ozawa

**Affiliations:** 1 Department of Health Policy and Management, UNC Gillings School of Global Public Health, University of North Carolina, Chapel Hill, NC USA; 2 Division of Practice Advancement and Clinical Education, UNC Eshelman School of Pharmacy, University of North Carolina, CB#7574, Beard Hall, 115H, Chapel Hill, NC 27599 USA; 3 Duke Global Health Institute, Duke University, Durham, NC USA; 4 Department of Pharmacy, School of Health Sciences, University of Zambia, Lusaka, Zambia; 5 Department of Epidemiology, Johns Hopkins Bloomberg School of Public Health, Baltimore, MD USA; 6 Malaria Research Institute, Johns Hopkins Bloomberg School of Public Health, Baltimore, MD USA; 7 Division of Clinical Pharmacology and Division of Infectious Diseases, Department of Medicine, Johns Hopkins University School of Medicine, Baltimore, MD USA; 8 Carolina Population Center, University of North Carolina, Chapel Hill, NC USA; 9 Department of Maternal and Child Health, UNC Gillings School of Global Public Health, University of North Carolina, Chapel Hill, NC USA

**Keywords:** Malaria, Antimalarials, Substandard, Falsified, Zambia, Quality

## Abstract

**Background:**

Many countries are striving to become malaria-free, but global reduction in case estimates has stagnated in recent years. Substandard and falsified medicines may contribute to this lack of progress. Zambia aims to eliminate their annual burden of 1.2 million pediatric malaria cases and 2500 child deaths due to malaria. We examined the health and economic impact of poor-quality antimalarials in Zambia.

**Methods:**

An agent-based model, Substandard and Falsified Antimalarial Research Impact (SAFARI), was modified and applied to Zambia. The model was developed to simulate population characteristics, malaria incidence, patient care-seeking, disease progression, treatment outcomes, and associated costs of malaria for children under age five. Zambia-specific demographic, epidemiological, and cost inputs were extracted from the literature. Simulations were run to estimate the health and economic impact of poor-quality antimalarials, the effect of potential artemisinin resistance, and six additional malaria focused policy interventions.

**Results:**

We simulated annual malaria cases among Zambian children under five. At baseline, we found 2610 deaths resulting in $141.5 million in annual economic burden of malaria. We estimated that elimination of substandard and falsified antimalarials would result in an 8.1% (*n* = 213) reduction in under-five deaths, prevent 937 hospitalizations, and realize $8.5 million in economic savings, annually. Potential artemisinin resistance could further increase deaths by 6.3% (*n* = 166) and cost an additional $9.7 million every year.

**Conclusions:**

Eliminating substandard and falsified antimalarials is an important step towards a malaria-free Zambia. Beyond the dissemination of insecticide-treated bed nets, indoor residual spraying, and other malaria control measures, attention must also be paid to assure the quality of antimalarial treatments.

## Background

Significant strides have been made to reduce the burden of malaria worldwide; yet global progress has stagnated in recent years [1]. The World Health Organization (WHO) estimates that nearly half of the world’s population is at risk for malaria, where children under age five are the most vulnerable to the disease [2].

In Zambia, an estimated 2.7 million cases of malaria due to *Plasmodium falciparum (P. falciparum)* occurred in 2018 [3]. Malaria disproportionately affects Zambian children under five with around 1.2 million pediatric cases and 2500 children dying annually [3–6]. Since 2006, the Zambian government has made a concerted effort to reduce the incidence of malaria. The burden of malaria declined until 2009, after which there was an annual increase in the number of estimated cases until 2015, when it started to fall again [1]. In 2017, Zambia launched its National Malaria Elimination Strategic Plan with the goal of a malaria-free Zambia by 2021 [6].

A key component of Zambia’s current multifaceted approach is to provide access to quality-assured antimalarial medicines [6], which are prequalified by the WHO prequalification programme or authorized for use by the Zambia Medicines Regulatory Authority (ZAMRA) [7]. However, not all outlets where antimalarials are procured stock solely quality-assured products [8]. In 2008, less than 5% of public facilities in Zambia were stocking non-quality assured artemisinin-based combination therapies (ACTs) [9]. Yet by 2014, non-quality assured medications rose to make up 31.6% of antimalarials stocked in public facilities in Zambia [8]. This leaves room for potential sale and purchase of substandard and falsified medicines or degradation of non-quality-assured products.

As defined by the WHO and for the purposes of this paper, substandard medicines are “authorized medical products that fail to meet either quality standards, specifications, or both,” and falsified medicines are medical products that “deliberately or fraudulently misrepresent their identity, composition, or source” [10]. Consuming substandard or falsified antimalarials can harm patients, leading to longer duration of illness, higher probability of severity, and greater selection for antimicrobial resistance. The economic consequences of these health effects impact patients through loss of income and payments for care induced by poor-quality medicines, and affect society through loss of productivity of patients who die prematurely or suffer from disability [11].

A recent meta-analysis revealed that 19.1% of antimalarials in low- and middle-income countries (LMICs) were substandard or falsified [12]. However, data specific to the prevalence of substandard and falsified antimalarials in many LMICs is minimal, making the use of simulation modeling important in understanding and assessing the burden resulting from use of these products. In a review of six sub-Saharan African nations not including Zambia, the WHO found that 28.5% of antimalarial samples were non-complaint with quality specifications (with non-extreme and extreme deviations) and 11.6% of antimalarials were found to have extreme deviations [13, 14]. In Zambia, one study found that 10.3% of antimalarials tested contained less than 80% of the labeled active pharmaceutical ingredient [15]. Moreover, through post-market surveillance at hospitals in three provinces, ZAMRA found in 2019 that 22 of 125 (17.6%) essential medicine samples including antimalarials did not pass the Global Pharma Health Fund’s Minilab tests [16, 17].

Previous analyses across Sub-Saharan Africa found that deaths due to substandard and falsified antimalarials comprise between 3.8 and 8.9% of malaria deaths relating to cases seeking treatment [18]. Country level analyses have demonstrated that substandard and falsified antimalarials contribute substantially to the pediatric malaria burden in both Nigeria and Uganda [19, 20]. While existing literature on antimalarials in Zambia emphasizes the cost-effectiveness of treating malaria with ACTs [21], to the best of our knowledge no study has yet examined the economic impact of substandard and falsified antimalarials in Zambia. This study attempts to understand the health and economic impact due to consumption of substandard and falsified antimalarials by children in Zambia.

## Methods

### Agent-based model simulation

The Substandard and Falsified Antimalarial Research Impact (SAFARI) model was developed to estimate the health and economic impact of substandard and falsified antimalarials among children under five by simulating population characteristics, malaria incidence, patient care-seeking, disease progression, treatment outcomes, and associated costs [19, 20, 22]. The SAFARI model is an agent-based model implemented in Python. We developed the SAFARI model and have applied it to other countries in Sub-Saharan Africa in the past [19, 20, 22]. Methods underscoring the SAFARI model have been published previously [19]. We outline the key methods here, with descriptions of the alterations and assumptions made for the Zambian context. The main demographic, epidemiological, and cost inputs are presented in Table 1.

**Table 1:** SAFARI Model Inputs for Zambia

**Model Input**	**Point Estimate**	**Range/SD**	**Source**
**Demographic & Epidemiological Data**			
< 5 Population At Risk	2,750,360		President's Malaria Initiative 2019 [6]
Untreated Case Progression to Severe	0.13	0.07–0.30	Lubell 2011 [23]
Treatment Failure Progression to Severe	0.02		Lubell 2011 [23]
Inpatient Case Fatality Rate	0.08		Camponovo 2017 [24]
Case Fatality Rate Outside of Hospital	0.15		Camponovo 2017 [24]
Inpatient Severe Case Rate of NS	0.032	0.0014	Lubell 2011 [23]
**Care-Seeking Behavior (%)**			
Public Facilities	67.5%		Zambian DHS 2018 [25]
Private Facilities	4.6%		Zambian DHS 2018 [25]
Pharmacies	0.3%		Zambian DHS 2018 [25]
Drug Stores	0.8%		Zambian DHS 2018 [25]
CHWs	3.0%		Zambian DHS 2018 [25]
Self/Neighbors	9.2%		Zambian DHS 2018 [25]
No Treatment	14.6%		Zambian DHS 2018 [25]
**Probability facility has antimalarial in stock**			
Public Facilities	99.8%		ACTwatch 2014 [8]
Private Facilities	90.5%		ACTwatch 2014 [8]
Pharmacies	88.8%		ACTwatch 2014 [8]
Drug Stores	92.1%		ACTwatch 2014 [8]
**Medication Effectiveness**			
ACTs Effectiveness	0.962	0.044	**Nambozi 2011, Bassat 2009, 4ABC 2011,** Hamainza 2014, Pekyi 2016, Chanda 2006, Depoortere 2005 [26–32]
SP Effectiveness	0.759	0.741–0.905	Depoortere 2005 [31]
Other Drug Effectiveness	0.88	0.041	Yeka 2013, Verret 2011 [33, 34]
**Substandard and Falsified Parameters**			
Proportion of SF Antimalarials (API > 85%)	0.103		Bate 2010 [15]
Increase in Probability of Transitioning to Severe Malaria if Receive SF Drug	50%		Assumption
**Treatment Adherence**		**Coefficient**	
Good: Probably Adherent	0.3940	1	Depoortere 2004 [35]
Fair: Perhaps Not Adherent	0.3940	0.5	Depoortere 2004 [35]
Poor: Definitely Not Adherent	0.2120	0	Depoortere 2004 [35]
**Patient Costs for Drugs**			
**Public Facilities**			
Average Costs of ACTs	$0.00		ACTwatch 2014 [8]
Average Costs of SP	$0.00		ACTwatch 2014 [8]
Average Costs of Other Treatments	$0.00		ACTwatch 2014 [8]
**Private Facilities**			
Average Costs of ACTs	$5.23	$2.09 - $12.81	ACTwatch 2014 [8]
Average Costs of SP	$0.65	$0.33 - $1.96	ACTwatch 2014 [8]
Average Costs of Other Treatments	$5.23	$0.00 - $8.24	ACTwatch 2014 [8]
**Pharmacies**			
Average Costs of ACTs	$5.23	$3.92 - $7.85	ACTwatch 2014 [8]
Average Costs of SP	$0.33	$0.26 - $0.65	ACTwatch 2014 [8]
Average Costs of Other Treatments	$5.49	$3.84 - $5.49	ACTwatch 2014 [8]
**Drug Stores**			
Average Costs of ACTs	$1.96	$1.31 - $3.14	ACTwatch 2014 [8]
Average Costs of SP	$0.39	$0.26 - $0.39	ACTwatch 2014 [8]
Average Costs of Other Treatments	$1.37	$0.00 - $0.00	ACTwatch 2014 [8]
**Other Costs to Patients**			
Transport Costs (Public, Private)	$2.51	$1.25 - $6.26	Aspler 2008 [36]
Transport Costs (Pharmacies, Drug Stores)	$0.25		Assumption based on: Aspler 2008 [36]
Costs of Special Food for Children	$3.12	$0.96 - $6.26	Aspler 2008 [36]
Costs per Pediatric Malaria Hospitalization	$10.56	$2.69	WHO CHOICE 2010 [37]
Productivity Loss per Sick Day	$3.21		Calculation based on World Bank 2019 [38]
Productivity Loss from Death, Discounted at 3%	$30,155.30		Calculation based on World Bank 2019 [38]
NS Disability Productivity Loss, Discounted at 3%	$12,477.37		Calculation based on World Bank 2019 [38]
**Facility Costs**			
Facility Costs per Testing	$0.96	$0.21	Chanda 2009 [39]
Facility Costs per ACTs	$2.00	$0.50	Chanda 2007 [40]
Facility Costs per SP	$0.27	$0.06	Chanda 2007 [40]
Facility Costs per Other Treatments	$0.11		Calculation
Public Facility Costs per Consultation	$6.40	$1.51	Chanda 2007 [40]
Facility Costs per Pediatric Malaria Hospitalization	$84.07	$6.72	Comfort 2014 [41]
Costs per CHW Treatment	$1.01	$0.19	Chanda 2011 [42]
Costs per CHW Testing	$3.14	$0.79	Haimanza 2014 [43]
Costs per CHW Visit	$3.32	$0.79	Chanda 2011 [42]

*ACTs* artemisinin-based combination therapies, *API* active pharmaceutical ingredient, *CHWs* community health workers, *DHS* demographic and health survey, *NS* neurologic sequelae, *SD* standard deviation, *SF* substandard or falsified, *SP* sulfadoxine-pyrimethamine, *WHO* World Health Organization

**Fig. 1:**
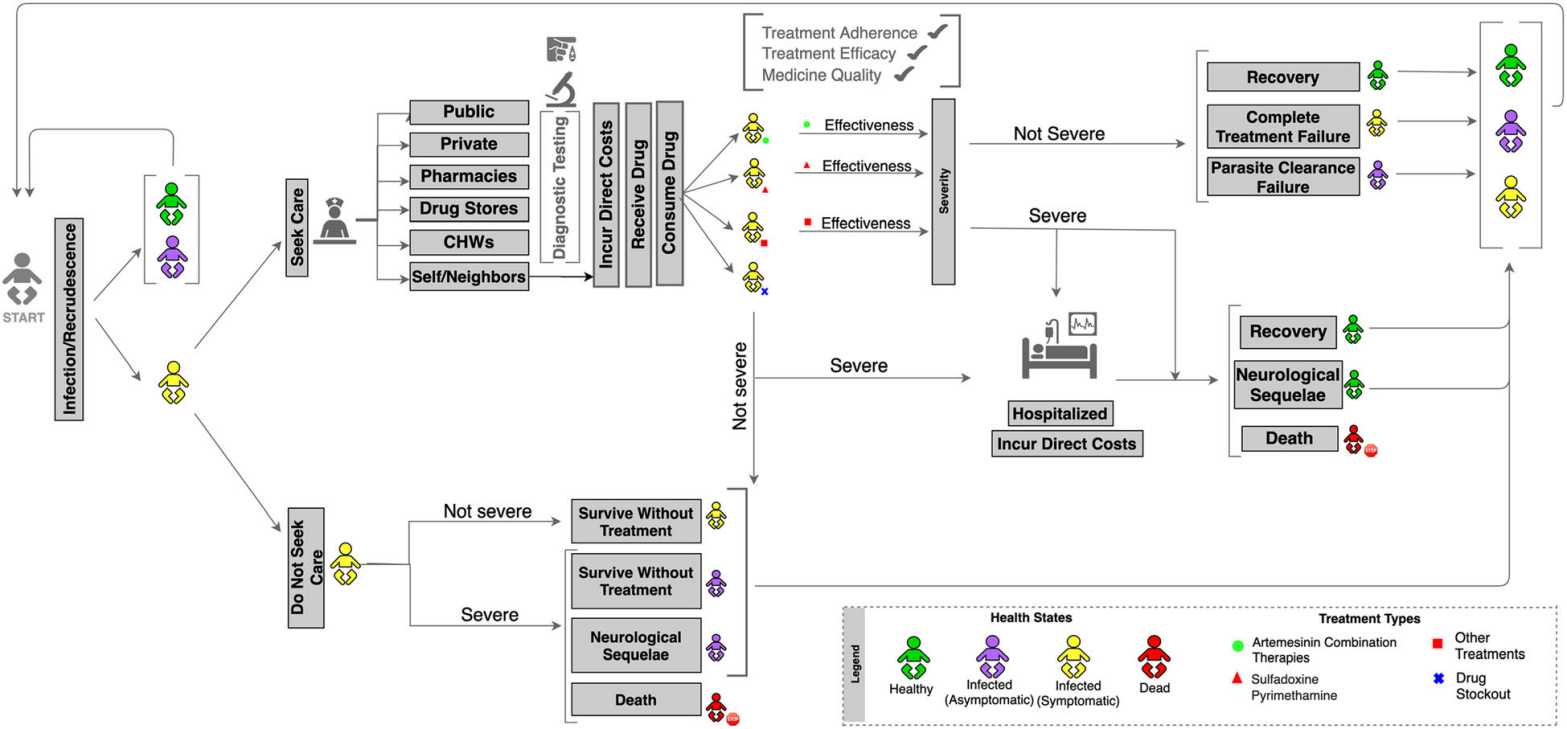
Flow Diagram of the SAFARI Model for Zambia

A flow diagram in Fig. 1 depicts the movement of child agents through the SAFARI model for Zambia. The model simulates 25,000 child agents over a one-year time horizon accounting for care-seeking and average duration of symptoms. The model is run in five-day increments to match the reported average duration of an uncomplicated *P. falciparum* malaria case. All child agents begin healthy and then move through the disease and care-seeking simulations. If the child agent becomes infected and symptomatic, the model simulates treatment at one of six care-seeking locations (public facility, private facility, pharmacies, drug stores, community health workers or self-medication including receiving medicines from friends/neighbors) or the progression of disease without seeking care.

Antimalarial treatment available in each location is simulated based on the market share of three medicines: ACTs, sulfadoxine-pyrimethamine (SP), or other treatments (e.g. quinine). In Zambia, 97.4% of the pediatric population receive ACTs and 1.3% receive SP and 1.3% received another drug (quinine, chloroquine, other antimalarial) for treatment of uncomplicated malaria based on data from the Demographic and Health Survey (DHS) [25]. The recommended treatment for pediatric malaria is artemether-lumefantrine branded as Coartem, which is available for free in public facilities. We simulated that each care location could run out of stock of antimalarials using facility specific probabilities of having ACTs in stock, derived from national stock availability data from ACTwatch [8]. At the end of a five-day period, child agents who are healthy, infected and asymptomatic, or infected and symptomatic return to the start of the model. Those who have progressed to a severe case face a probability of death according to case fatality rates from the literature and are removed from the model simulation, or face a probability of suffering from neurological sequelae [23, 24].

### Demographic, epidemiologic, and cost data inputs

One of the benefits of agent-based models is the ability to assign demographic characteristics, individual incidence, and individual care-seeking rates to each child agent. Demographic characteristics such as age, socio-economic status, mother’s education, urban/rural residence, and region (zones) were assigned to child agents based on the reported national prevalence in Zambia, retrieved from the 2018 Zambia DHS and the 2018 Malaria Indictor Survey (MIS) [25]. Individual incidence and care-seeking probabilities were calculated for each child agent based on the combination of these characteristics, allowing for variation in the model and facilitating outputs to be examined on a more granular level.

Electronic databases (PubMed, Google Scholar, and EBSCOhost) were searched for published literature related to antimalarials, medicine quality, pediatric malaria, and costs of care to identify model inputs specific to Zambia. Gray literature was used to isolate additional inputs from sources such as ACTwatch, the Zambia DHS/MIS, the World Malaria Report, and the Worldwide Antimalarial Resistance Network (WWARN) [1, 8, 25, 44]. In order to account for the natural variation in epidemiologic and cost inputs, these inputs were sampled from specified distributions using means and standard deviations from the literature across 10,000 model runs. Cost inputs were ranged across beta distributions and epidemiological inputs were ranged using gamma distributions. All costs are reported in 2018 USD ($). Costs in Zambian Kwacha were inflated from various years to 2018 Kwacha using Zambia’s inflation rates and converted to 2018 USD using exchange rates from the U.S. Treasury [45, 46].

Treatment efficacy for each antimalarial and prevalence of substandard and falsified medicines for Zambia were obtained via the WWARN database [26–34, 44]. Due to a small number of available studies on efficacy of quinine in children under five in Zambia, regional studies were utilized for child agents who received treatments other than ACTs or SP (approximately 2.9% of cases) [32–34]. To account for the increase in adverse outcomes caused by substandard or falsified antimalarials, it was assumed that patients who received poor-quality antimalarials faced a 50% increase in the probability of developing severe malaria within a week of receiving treatment, reflecting the impact of reduced efficacy of antimalarials with lower amounts of active pharmaceutical ingredients [18]. Each child agent in the model was assigned a probability of treatment adherence, which affected treatment success [35]. Treatment outcomes for each child agent in the model were determined based on individual treatment adherence rates, treatment efficacy by medication, and quality of obtained antimalarials.

### Health and economic outputs

Primary outputs of the model are estimates of the health impact, direct costs, and productivity losses attributable to substandard and falsified antimalarials among children under five in Zambia. The health impact is presented as numbers of uncomplicated and severe cases, cases of neurological sequelae, and deaths due to malaria. Economic outputs report direct costs, which include costs for transportation, testing, drugs, consultation, and hospitalization, as well as productivity losses [23, 36, 37, 39–43]. Productivity losses are calculated from Zambia’s Gross Domestic Product (GDP) per capita, and are broken into short-term losses, which include time lost by care-takers, and long-term losses over a lifetime due to children suffering from malaria-induced disabilities or premature deaths [38]. Future long-term productivity losses were discounted at 3%. At public facilities, direct costs were further separated into those paid by patient families out-of-pocket versus those incurred by public facilities.

### Scenario analyses

We analyzed the effect of substandard and falsified antimalarials among the under-five population by simulating three antimalarial quality scenarios. First, we estimated the burden of malaria in Zambia and utilized this scenario as our baseline estimate. We then isolated the effect of substandard and falsified antimalarials on the health and economic impact of malaria that is due to 10.3% prevalence of poor-quality antimalarials [15]. In order to address the uncertainty around the prevalence of substandard and falsified antimalarials, we also examined a scenario in which Zambia experienced 19.1% prevalence of substandard and falsified antimalarials, which is the average across all LMICs [12]. These two scenarios (10.3 and 19.1% prevalence) provide a range for the possible impact of poor-quality antimalarials in the country, given the limited evidence on the prevalence of substandard and falsified antimalarials in Zambia. We additionally examined the hypothetical implications of *P. falciparum,* the parasite causing almost all malaria cases in Zambia, potentially developing resistance to ACTs. To simulate this scenario, we reduced the effectiveness of ACTs to that of less effective treatment (SP), and prolonged the illness for each child agent taking ACTs by 5 days, to reflect the increase in illness duration likely associated with antimalarial resistance [47]. This scenario was compared to the baseline scenario to estimate the additional impact of potential antimalarial resistance.

Lastly, we implemented six scenario analyses simulating proposed interventions to assess their impact on malaria outcomes in Zambia. These scenarios included: 1) no antimalarial stock-outs in any facilities; 2) no antimalarial stock-outs in public facilities; 3) no antimalarial stock-outs in private facilities; 4) replacement of other treatment alternatives with ACTs; 5) increasing the percentage of individuals seeking health care by 20% among those currently not seeking care; and 6) having no substandard or falsified antimalarials. Each scenario was examined separately, holding all other inputs constant as baseline, in order to understand the impact of the policy options.

## Results

Findings on the health and economic impact of substandard and falsified antimalarials in Zambia are summarized in Table 2. With an under-five population of 2.8 million in Zambia [48], the baseline model simulated 1.2 million cases of malaria annually in Zambian children under five. Of the total cases, we simulated that 11,071 cases (95% confidence interval (CI) 11,039 – 11,103) resulted in hospitalizations, 295 cases (95% CI 291–299) suffered from neurological sequelae, and 2610 cases (95% CI 2598 – 2622) resulted in death, annually. The total estimated annual economic impact of malaria in children under-five in Zambia was $141.5 million (95% CI $141.0 – $141.9 million), with $114.6 million (95% CI $114.2 – $115.0 million) attributed to productivity losses, and $11.7 million (95% CI $11.5 – $11.9 million) in direct costs for health care.

**Table 2:** Comparison of the Health and Economic Impact of Scenarios including SF Antimalarials

	Total Burden of Malaria with an SF Prevalence at 10.3%		Savings by eliminating 10.3% prevalence of SF Antimalarials		Total burden of SF Antimalarials with SF Prevalence of 19.1%		Antimalarial Resistance Scenario	
	Burden	95% CI	Impact	%	Impact	%^a^	Impact	% Diff.
Average cases	1,216,454	(1,216,231 - 1,216,676)						
Average deaths	2610	(2598 - 2622)	213	8.2%	365	13.2%	166	6.3%
Average hospitalizations	11,071	(11,039 - 11,103)	937	8.5%	1643	14.0%	1048	9.5%
Average cases of NS	295	(291 - 299)	9	3.1%	11	3.8%	19	6.5%
Total economic impact	$141,470,907	(141,037,214 - 141,904,600)	$8,541,887	6.0%	$14,465,329	9.8%	$9,662,235	6.8%
Facility costs	$15,174,357	(15,134,100 - 15,214,614)	$$411,098	2.7%	$637,725	4.1%	$1,266,646	8.3%
Out-of-pocket costs	$11,699,978	(11,536,334 - 11,863,623)	$423,151	3.6%	$478,061	4.1%	$677,144	5.8%
Total productivity losses	$114,596,572	(114,200,504 - 114,992,639)	$7,707,639	6.7%	$13,349,543	11.1%	$7,718,445	6.7%
Short-term productivity losses	$25,162,247	(25,090,781 - 25,233,713)	$531,091	2.1%	$1,021,167	4.0%	$2,053,261	8.2%
Total lifetime productivity losses	$89,434,325	(89,044,819 - 89,823,831)	$7,176,548	8.0%	$12,328,376	13.0%	$5,665,184	6.3%

*CI* confidence interval, *NS* neurologic sequelae, *SF* substandard and falsified

^a^Percentage of the total burden of malaria when SF prevalence is 19.1%

Across 10,000 model runs, the burden of malaria attributable to substandard and falsified antimalarials was estimated at 213 (95% CI 212–213) deaths and 937 (95% CI 937–938) child hospitalizations every year, given a 10.3% prevalence of substandard and falsified antimalarials. In other words, if substandard and falsified antimalarials are replaced by high-quality ones, Zambia could experience an 8.2% reduction in under-five deaths (213 fewer deaths), observe a 8.5% reduction in the number of hospitalizations (937 fewer hospitalizations) and realize $8.5 (95% CI $8.5 – $8.6) million in total economic savings including averting direct costs to patients and the government, as well as preventing productivity losses due to death or life lived with disability.

Given the uncertainty in the prevalence of substandard and falsified medicines in Zambia, we simulated a further scenario in which the prevalence of poor-quality antimalarials in Zambia was as high as the LMIC-wide average of 19.1%. In this scenario, a significantly higher health and economic impact was found to be attributable to poor-quality antimalarials. Annually, a 19.1% prevalence of substandard and falsified antimalarials would result in $14.5 (95% CI $14.5 – $14.5) million in costs, lead to 365 (95% CI 365–366) under-five deaths, and be responsible for 1643 (95% CI 1643 – 1644) pediatric hospitalizations.

We also examined the hypothetical impact of antimalarial resistance in Zambia. In the event that effectiveness of ACTs, the recommended treatment for malaria, is reduced to that of other treatments with less effective antimalarials (SP) and prolonged duration of illness, we simulated that Zambia would see an increase of 166 (95% CI 165–166) deaths (6.3%), and 1048 (95% CI 1048–1048) under-five hospitalizations (9.5%), annually. The resulting economic impact would be $9.7 (95% CI $9.6 – $9.7) million per year, with $7.7 (95% CI $7.7 – $7.7) million attributed to productivity losses.

Table 3 presents the results of six intervention scenarios, which improve stock-outs, antimalarial quality, and access to care. We found that removing substandard and falsified medications (assuming prevalence at 10.3%) had the second-greatest impact, following increasing the number of children who seek care for malaria. If 20% of children who did not seek care for malaria sought care, we simulated economic savings of $12.7 (95% CI $12.7 – $12.7) million, assuming that the supply of antimalarials can rise with increased demand, maintaining the same proportion of quality-assured antimalarials. We estimated that removing stock-outs had a smaller effect than improving the quality of antimalarial medications. If stock-outs were eliminated in both the public and private sector maintaining the same proportion of quality-assured vis-à-vis non-quality assured antimalarials, this is estimated to reduce under-five deaths by 5.4% (141 fewer deaths; 95% CI 141–141) and result in economic savings of $5.5 (95% CI $5.5 – $5.5) million annually.

**Table 3:** Impact of Intervention Scenarios Compared to Baseline

	Deaths	Total Economic Impact		Total Productivity Losses	
		Estimate	95% CI	Estimate	95% CI
Baseline	2610	$141,470,907	(141,037,214 to 141,904,600)	$114,596,572	(114,200,504 to 114, 992,639)
No SF Antimalarials^a^	−213	−$8,541,887	(−$8,547,760 to −$8,536,014)	−$7,707,639	(− $7,713,090 to −$7,702,188)
No stock-outs all outlets^b,c^	−141	−$5,471,452	(−$5,490,113 to −$5,452,791)	−$5,092,216	(−$5,109,558 to −$5,074,874)
No public stock-outs^c^	−69	−$2,920,369	(−$2,926,294 to −$2,914,444)	−$2,534,053	(−$2,539,559 to −$2,528,548)
No private stock-outs^c^	−18	−$729,901	(−$735,857 to −$723,945)	−$584,179	(−$589,739 to −$578,620)
Only ACTs are available^d^	2	−$56,718	(−$62,749 to −$50,688)	$85,065	($79,476 to $90,654)
Increase in care seeking habits^c,e^	−345	−$12,713,276	(−$12,719,093 to −$12,707,459)	−$12,466,581	(−$12,471,928 to −$12,461,234)

*ACTs* artemisinin-based combination therapies, *CI* confidence interval, *SF* substandard and falsified

^a^Simulated the prevalence of substandard and falsified antimalarials at 10.3%

^b^This includes public and private hospitals, pharmacies, general retailers, and drug stores

^c^These scenarios assumed that supply of antimalarials would increase to meet the demand, keeping the proportion of substandard and falsified antimalarials and the mix of available antimalarials the same as baseline

^d^This scenario was not statistically significantly different from baseline given high ACT availability at 97.4%

^e^20% of those who did not seek care for child malaria were assumed to seek care

## Discussion

Our SAFARI model results highlight the importance of eliminating substandard and falsified antimalarials in Zambia to reach the goal of malaria elimination by 2021. Compared to the LMIC-wide average prevalence of substandard and falsified antimalarials (19.1%), our results indicate that maintaining a lower prevalence (10.3%) of substandard and falsified medicines would mean a difference of 153 lives and $5.9 million in costs annually in Zambia. Based on the model, eliminating all substandard and falsified antimalarials in Zambia will result in an additional $8.5 million in economic savings, 937 fewer hospitalizations, and save 213 children’s lives every year.

At present, 80.6% of households in Zambia have access to at least one form of vector control, involving indoor residual spraying or insecticide-treated bed nets [5]. While vector control programs can reduce the incidence of malaria, access to quality-assured antimalarials is essential to stop the spread of disease when people acquire malaria. Zambia currently uses the WHO medicine prequalification programme for quality-assured medicines [49]. ZAMRA also conducts random testing of medicines through post-market surveillance using the Global Pharma Health Fund’s GPHF-Minilab kits, and removes suspected substandard or falsified medicines off the market for further testing [16, 17, 50]. Wider access and use of cost-effective detection devices for substandard and falsified medicines can mean detecting and removing poor-quality antimalarials more effectively.

Zambia has succeeded in centralizing access to and incentivizing the purchase of antimalarials via public facilities, but improved surveillance methods are needed throughout the supply chain to quickly identify and remove substandard and falsified antimalarials. As a landlocked country with many borders, Zambia is especially vulnerable to importation of substandard and falsified medicines. Establishing a coordinated and strategic surveillance program can prevent harm from the use of poor-quality medical products. Surveillance in the private sector should be strengthened as patients can access antimalarials through privately-run retail stores, community pharmacies, private hospitals, and private clinics.

Our model estimates that reduction of stock-outs in the public and private sector would result in 141 fewer deaths (5.4% reduction) and avert 133 hospitalizations (1% reduction), assuming current proportions of quality-assured antimalarials are made available to fill the gap in supply. Stock-outs of quality-assured ACTs in the public sector can result in patients seeking medication from alternative retailers who stock cheaper medications, such as non-ACTs and possibly poor-quality antimalarials [49]. Presence of unlicensed drug stores increases the risk of having poor-quality medicines in the market, as it is illegal for these stores to obtain medicines from registered wholesalers. Instead, these stores commonly acquire medicines from unregistered or cross-border vendors.

Since Zambia currently imports all of their medications, it can be challenging to meet the local demand for antimalarials. Fees charged by ZAMRA to import medicines are high – $2000 and $2800 per product for generic and innovator brands, respectively, and $4000 to fast track medicine authorization. Those fees, along with the presence of sole distributorship arrangements, are perceived as barriers to increasing the supply of antimalarials and contribute to increased stock-outs [49]. Producing high-quality medication in Zambia may be one solution to this and potential policies have been put forward that seek to support the development of Zambian medicine manufacturing. This idea has traction in Zambia where four such facilities have opened in the past 3 years. Ensuring good manufacturing practices to generate quality-assured products is essential across both imported and domestically manufactured medicines.

Access to care continues to be a challenge in rural Zambia, with only 42% of caregivers reporting to seek early treatment within 24 h for children with fever [51]. Improving access to care is important for reducing the burden of malaria, as seeking care earlier reduces the likelihood of complications and death. Increasing care-seeking by, for example, implementing Universal Health Coverage (UHC) could result in higher use of quality-assured medicines overall, if measures to assure the quality and quantity of additional medicines are also implemented. This implies that greater numbers of people will seek care through the formal system, where medicine quality could be regulated.

At the same time, increased care-seeking can put a strain on existing systems, increasing demand for medicines sought within the system, and requiring additional resources. Our simulation of increased care-seeking assumed health systems capacity exists to treat additional patients at current levels of care but that other barriers to access care remained [52]. In order to see improvements from care-seeking, availability of quality-assured medicines needs to increase accordingly. If the stock of medicines would not adjust with increases in care-seeking, we would expect that the benefits of increased care-seeking would be much lower, as stock-outs would worsen. Increasing care seeking and medicine utilization in a system that is not prepared could in fact worsen medicine quality if producers lower quality to stay within profit margins [53]. Thus, the benefits of the increased care-seeking scenario would only materialize if the health system and supply of quality-assured medicines can accommodate increases in demand.

Our baseline results are similar to past estimates of under-five malaria disease burden in Zambia. Our model simulates 1.2 million under-five cases annually, which is comparable to the President’s Malaria Initiative estimate that 35% of all malaria cases (3.5 million in Zambia) occur in children under five [1, 6]. We chose to calibrate the model to the estimated number of cases from the World Malaria Report, which provides a more conservative estimate than alternatives that convert parasitic rates to case incidence [1]. Furthermore, our model estimates 2610 under-five deaths due to malaria annually, which is similar to the United Nations Children’s Fund’s (UNICEF) estimates of 2558 annual deaths in 2016 [4].

There are several limitations to note. First, the quality and availability of data surrounding antimalarial quality in Zambia is limited, which made it difficult to capture the heterogeneity within Zambia [54, 55]. We incorporated the most recent reliable data available at this time by conducting literature searches for model inputs. For inputs where Zambian pediatric malaria data were not available, Zambian adult inputs or pediatric inputs from neighboring countries were used. We address uncertainty around data inputs by probabilistically ranging variables in the model, and recording ranges across 10,000 model runs. Moreover, the prevalence of substandard and falsified medicines came from a single study in Zambia, which tested few antimalarial samples [15]. Further studies are needed to more effectively capture the current prevalence of poor-quality medicines in Zambia using rigorous sampling and testing methods. Lastly, our study focused on the economic costs of substandard and falsified medicines rather than the costs of interventions to improve supply chains or strengthen health systems. Such analyses could be helpful to estimate the cost-benefit ratios of medicine quality interventions. Despite these limitations, we believe our health and economic impact estimates are vital to raising public awareness of the effects of substandard and falsified antimalarials in Zambia.

## Conclusions

The presence of substandard and falsified malaria medications worldwide along with the stagnation in malaria case reductions threaten global health security. This study shows that reduction of substandard and falsified antimalarials could substantially help Zambia reach the goal of malaria elimination. Zambia has successfully increased malaria prevention measures including the adoption of insecticide-treated bed nets and indoor residual spraying, but attention must also be paid to assuring antimalarial quality within the country. Increased funding and capacity building of ZAMRA and close coordination with the Ministry of Health is important to monitor and regulate the medicines supply chain, remove substandard and falsified antimalarials, and reduce stock-outs. Failing to do so would likely result in increases in malaria mortality and morbidity.

## Data Availability

All data utilized during this study were taken from publicly available data and are included in this published article.

## Abbreviations

ACTs: Artemisinin-based Combination Therapies
DHS: Demographic and Health Survey
GDP: Gross Domestic Product
LMICs: Low- and Middle-Income Countries
MIS: Malaria Indicator Survey
SAFARI: Substandard and Falsified Antimalarial Research Impact
SP: Sulfadoxine-Pyrimethamine
UNICEF: United Nations Children’s Fund
USD: United States Dollars
WHO: World Health Organization
WWARN: Worldwide Antimalarial Resistance Network
